# Youth political participation and digital movement in Indonesia: the case of #ReformasiDikorupsi and #TolakOmnibusLaw

**DOI:** 10.12688/f1000research.122669.3

**Published:** 2024-04-25

**Authors:** Sri Lestari Wahyuningroem, Rheinhard Sirait, Uljanatunnisa Uljanatunnisa, Dudy Heryadi

**Affiliations:** 1 Political Science, Universitas Pembangunan Nasional Veteran Jakarta, Jakarta Selatan, DKI Jakarta, 12450, Indonesia; 2 Communication Science, Universitas Pembangunan Nasional Veteran Jakarta, Jakarta, DKI Jakarta, 12450, Indonesia; 3 International Relations, Universitas Padjadjaran, Bandung, West Java, 45363, Indonesia

**Keywords:** youth, political participation, digital movement, democracy

## Abstract

The younger generations have always been at the forefront of and contributed significantly to social movements that are critical of state policies or respond to socio-political situations. This is also what happened in large movements involving young people in Indonesia, conventionally in the form of demonstrations and digital movements. This study seeks to explore the relationship between youth political participation, digital media, and the development of youth political identity by analyzing young people’s engagement in some of the most significant political protests in Indonesia during the mass protests of #ReformasiDikorupsi, a movement opposing the amendments of several important bills, and #TolakOmnibusLaw, a movement opposing the establishment of a new single bill related to labor and investment, between 2019 and 2020. By analyzing quantitative data through data mining from Twitter from October 2019 to November 2020 with the *social network analysis* method, this study discusses online youth participation in social movements and the way they shape their political identity. Our research found that young people play an active and central role in building online and offline movements. In particular, the online movement contributes to the growth of the offline protest movement, and vice versa, because of concerns about the practice of illiberal democracy.

## Introduction

The Internet and social media have greatly affected the participation of student movements in digital democracy in Indonesia; numerous protests have been carried out against corruption, and changes in democratic patterns ( Saud & Margono, 2021), as seen in the #ReformasiDikorupsi movement in 2019 and #TolakOmnibusLaw movement in 2020. #ReformasiDikorupsi (Corrupted Reform) was a protest against the ratification of the Draft Law (RUU) of the Corruption Eradication Commission (KPK), which is considered likely to curb civil liberties and freedom of expression ( Sastramidjaja, 2020), while #TolakOmnibusLaw was a 2020 movement of students, trade unions, and civil society groups fighting against or rejecting the Omnibus Law on Job Creation, which was considered detrimental to workers ( Sastramidjaja & Rasidi, 2021). This study will focus on the relationship between youth political participation, digital media, and the development of youth political identity by analyzing young people’s experiences of participation in Indonesia, especially during the mass protests of #ReformasiDikorupsi in 2019 and #TolakOmnibusLaw in 2020.

Recent studies indicate that the state of democracy in Indonesia has experienced ups and downs in a situation that some scholars call illiberal democracy ( Hadiz, 2017; Diprose *et al.*, 2019). This condition is characterized as blocking citizens from obtaining knowledge about the activities of power holders due to the lack of civil liberties, despite holding elections. Closed legislation processes against various laws that are considered detrimental to general society have triggered large-scale demonstrations, primarily attended by workers, university students, high school students, and other various groups of youth. The protests came in distinct waves. The first wave was seen in late 2019 when the government and parliament revised the criminal code and passed a law that reduced the independency of the Corruption Eradication Commission (KPK), an Indonesian institution that had revealed a number of high-profile corruption cases. The second and much larger wave was observed in October 2020, protesting against the Omnibus Law on Job Creation.

In these political events, the role of young citizens was not only participants but also core actors who mobilized these protest movements to become so large-scale. We refer to young people as a diverse and heterogeneous societal group showing various complex identities—psycho-social, politico-economic, and educational. Indeed, young people are not a monolithic group whose members all feel and want the same or have convergent interests. This study particularly focuses on young people between the ages of 15 and 30 years. The UN Secretariat uses the terms “youth” and “young people” interchangeably to mean people aged 15–24 years with the understanding that member states and other entities use different definitions. In the Indonesian context, the category of young people is legally defined according to the 2009 Law of Youth; “youth” (Pemuda) as “Indonesian citizens who are entering an important period of growth and development and are aged between 16 and 30 years.” The 2020 national census reported that the number of youth is 75.49 million, or around 27.94 percent of the population ( BPS, 2021).

Many scientists have carried out studies on youth participation and digital society in the last two decades. Social and political dynamics in several countries are influenced by youth participation in the digital world; not infrequently, youth have been the driving force behind many social movements in various parts of the world. In this context, youth activism in the digital world is a consequence of the increasing loss of young people’s trust in conventional political models such as political parties and elections. In the United States, for example, a poll conducted by the Harvard Institute of Politics indicated consistently declining levels of trust in government institutions among Americans aged 18- to 29-year-olds between 2010 and 2015 ( Graeff, 2016, p. 95). In Europe, in 2013, the London School of Economics (LSE) Enterprise conducted a study on young people aged 16- to 26-year-olds who participated in voting and institutional politics. They reflect that “the political ‘offer’ does not match their concerns, ideas, and ideals of democratic politics.” At the same time, high levels of youth participation in issue-oriented activism, boycotting and buycotting, and protest activities were observed ( Graeff, 2016, p. 95). W. Lance Bennett describes this new generation of young people as “actualizing citizens,” “who favor loosely networked activism to address issues that reflect personal values,” in contrast with “dutiful citizens,” who maintain more collective and government-centered practices (Bennet, quoted in Graeff, 2016, p. 96).

According to Mei (2021), youth involvement in digital activism is generally influenced by three factors: social identity, a desire to be heard, and an increase in online activism. Youth derive their social identity from their group membership and eating outside, and with protests, their identity becomes politically potent, as in the case of the 2010 Arab Spring protests. Furthermore, they are attracted to political action because they desire to be adequately recognized in the political environment. If not, the youth will find a unique method to express their voice. Moreover, the rise of online activism through social media has dramatically influenced young people to become more politically involved and aware, as demonstrated in the #BlackLivesMatter movement in the United States, the #UmbrellaMovement in Hong Kong, and the #TeaMilk in Thailand.

Similar global research has considered Afghan youth’s political participation on Facebook ( Orfan, 2021). This study examines the effects of Facebook use on youth political involvement or participation in the 2019 presidential election. The results show that many Afghan youths needed to actively participate in Facebook. Another study by Sairambay (2021) examines the contributions of new media to young people’s political participation in Russia and Kazakhstan, showing that new media contributed to political participation in Russia and Kazakhstan among young people both online and offline through raising awareness as a prerequisite for political participation, and supporting communication, interaction, advocacy as political participation, mobilization, organization, and coordination.

In the local context, research on the rise of digital democracy and youth’s political participation in Indonesia by Saud and Margono (2021) is noteworthy. The study focuses on the influence of digital media on young people’s political involvement through digital platforms during the student movement in 2019 as a response to the ratification of the Corruption Law (KPK). This study demonstrates that social media platforms increase the influence of young people in politics in Indonesia. Sastramidjaja (2020) studied the #ReformasiDikorupsi protest, claiming it to have opened a path for digital activism in Indonesia. The Internet, especially social media, has broken down communication barriers, and, accordingly the dissemination of information has become rapid and inclusive. Indonesian society has seen increased involvement of millennials and generation Z in digital activism because these two generations grew up with advanced technology. The corrupted reform movement has given a rise to a new “generation” of activism in Indonesia. This suggests that conventional activism in Indonesia has succeeded in shifting to digital activism, which holds new hope for democracy in Indonesia.

In another article, Sastramidjaja and Rasidi (2021) discuss conflicts centered around the Omnibus Law or the Job Creation Act (#TolakOmnibusLaw), both in direct action and virtual action, between mainstream media and social media, and between physical repression and control over cyberspace. Resistance to the Omnibus Law continued as of May 2020 in the public sphere, with direct action against the law and the trending hashtag #TolakOmnibusLaw on social media. The collective action against the Omnibus Law spread the issue and popularized several hashtags so that the discourse on Omnibus Law was not limited to netizens involving social media. Several mainstream media also reported the rejection of the Omnibus Law by referring to Twitter trends as an indicator of public voices. The community’s immediate rejection of the Omnibus Law has prompted the government to react by promoting several hashtags, such as #RUUCiptakerLindungiPekerja, through buzzers and several influencers. Buzzers are not only mere buzzers on social media but also have been persecuting netizens who are vocal about the Omnibus Law issue. This caused fear among netizens to raise their opinions in social media.

Both articles ( Sastramidjaja, 2020; Sastramidjaja & Rasidi, 2021) show the importance and relevance of looking at the trends of digital social movements, especially in the context of Indonesia, and in particular at the two movements that are the focus of this article. However, they did not specifically address the involvement of young people in the movement, nor did they capture the relevance of their participation in the analysis of weakened democracy in Indonesia. In this study, we will fill this gap and examine how their movements contribute to the political identity of young people.

Within the two movements featured in this study, the central part of the strategy adopted by young people is to take advantage of digital media platforms. We will specifically look at this participation on the Twitter platform. Twitter is an important medium in Indonesia because it is the most widely used to voice political aspirations as well as disseminate socio-political news. This is also supported by the fact that Twitter users in Indonesia are the fourth largest in the world at 25.25 million users as of July 2023 ( Annur, 2023). The key questions posed in this study are: What is the involvement and role of young people in digital-based social movements, and how are they related to conventional (offline) protests? How does weakening democracy, characterized by non-transparent and violent practices in handling criticism, contribute to youth engagement on social media? While governments, technology, and forms of technology and participation change over time, this study finds that increasingly mediated, connected, and participatory social lives occur in countries where democracy is challenged. The study seeks to identify the basic features and implications of youth politics—illiberal practices. This research contributes to studies in political science, especially related to social movements, digital politics, and youth politics. In particular, this research will provide new insights from the study of contemporary Indonesian politics.

## Literature review

We reviewed a number of literatures related to (1) digital activism, and (2) the link between online digital participation and conventional offline movement mobilisation.

*Digital activism*

To better understand political participation in social media, Paskarina (2020) explored digital activism and democracy, identifying six main points of digital activism practices standard in Indonesia: 1. Strategies to mobilize support or form social or political movements; 2. public spaces to discuss marginalized issues; 3. criticism and control of the government; 4. “hoaxtivism” and counter-hoaxtivism; 5. hacktivism; and 6. the process of building identity. According to the author, these six digital activism practices reflect how technology shapes the breadth of activism. This explains that advances in media technology are shaped by social, political, and economic needs and practices that drive digital activism. The digital activism we discuss in this paper, therefore, concerns the four forms of activism: 1. strategies to mobilize support or to form social or political movements, 2. creating a public space to discuss marginalized issues, 3. criticism and control of the government, and 4. the process of building identity. For the context of the two movements in Indonesia that we examine, the strategies and approaches are adopted from a similar movement in Hong Kong called the Umbrella Movement in 2014.

Agur and Frisch (2019) discuss how activists used social media as a component of digital activism during the 2014 Hong Kong protests and the extent to which social media influenced the success of these actions. Social media play a role in mobilizing, organizing, and inviting the public to be involved in the action. Agur and Frisch argue that social media played a role in measuring the sentiments of activists and the public and as a place to plan strategies to increase pressure on the government. Social media are also crucial in gathering protesters from various regions, as well as a place to avoid police accusations and facilitate rescue and advocacy if participants are arrested by the police. However, this study does not explicitly discuss the role of youth in the movement.

Wong (2021) examines, more specifically, youth involvement in the movement to explain how social media affects youth participation and outlines four key findings. First, the dichotomy between loyal and materializing citizens is an oversimplified description of the impact of social media on youth participation. Second, its actual impact depends on many mediating and contextual factors. Third, media use, particularly reliance on social media to receive and share news and information about public affairs, creates an echo chamber effect that significantly separates the opinions of young people engaged in social media from the rest of the population. Fourth, while changing the nature of traditional social movements, social media emphasizes personalization, blurs the lines between leaders and advocates, and bridges the connections between institutions and participants. The weakening of the traditional structures has created a new logic of cohesive behavior in political participation.

*Linking online and offline activism*

In the current study, we examined youth participation in digital activism and conventional (offline) activism. Online activism is no substitute for offline activism, as Baybars-Hawks says the two may work together for greater impact ( Baybars-Hawks, 2015). Cammaerts (2015) assumes that the use of information and communication technology, which includes activist communicative practices, can be divided into two categories: practices supported by social media and practices based on social media. As explained by Cammaerts (2015), in communicative practices supported by internet communication technology and social media, social media support many activities such as internal organization, recruitment, building networks, and mobilizing efforts, as well as coordinating direct actions that activists will carry out. Social media are independently capable of disseminating a range of movements without relying on mainstream media, as well as affording space for discussions, debates, and deliberation in decision-making as a forum for activists ( Cammaerts, 2015).

According to Cammaerts (2015), social media have a more constitutive nature. The Internet and social media are used by activists to fight ideological enemies, facilitate the surveillance of supervisors (sousveillance), and store protest artifacts ( Cammaerts, 2015). Meanwhile, the function and role of external social media, as Cammaerts (2015) puts, can involve three activities: First, mobilization and recruitment for direct offline action are more flexible and have a lower cost. Second, the establishment of independent alternative communication channels via social media allows activists to carry out self-mediation by distributing goals and framing movements more easily. As explained by Cammaerts (2015), self-mediation is done by distributing the framing and interpretation of the movement toward an issue through independent communication channels such as social media. Social media have given activists the ability to transmit text and visual discourses. They are also considered to have the potential to provide opportunities for communities and subordinate groups to build alternative collective identities. An essential part of the self-mediation process, according to Cammaerts (2015), is the production of protest artifacts by participants documenting their protest actions and distributing them through social media to expand the archives and self-representation of the protest actions carried out. The third activity is the practice of resistance, as activists use the Internet and social media as weapons against the enemy. Social media serve as an instrument for direct action such as sousveillance—supervision of supervisors or management from the bottom up.

## Methods

This study used a mixed-method approach to comprehensively collect and analyze case study data by integrating complementary quantitative and qualitative approaches ( Tashakkori & Creswell, 2007). The research data were digital conversations produced and mobilized by netizens on Twitter within the conversation period from October 2018, when the #ReformasiDikorupsi started, until the end of November 2019, after the #TolakOmnibusLaw movement ended. Conversations on Twitter are a form of cross-geographical dissemination of microblog information that is useful for researchers as a source of research data whose context can be seen ( Letierce *et al.*, 2010) and helps in predicting the dynamics of the data to some extent ( Eysenbach, 2011). This research quantitatively used text mining, machine learning word clouds, and social network analysis (SNA) through Gephi open-source software ( www.gephi.org) with specific computational configurations to produce quantified text data. Open source software (OSS) is a public good and alternative programming operating system that users can use, modify and develop mutually according to their respective data processing needs ( Kollock & Smith, 1999; Grodzinsky *et al.*, 2003). The quantified data are checked and re-verified by human-led annotation or manual data classification by humans.

This research started with a preliminary study in October 2018 that determined the accounts and hashtags relevant to the research needs of analyzing and describing the conversations of netizens on the Twitter platform about the issue of the Job Creation Law (Omnibus Law). This process also identified certain representative accounts and hashtags to produce conversations about the issues to be researched. These representative hashtags constituted the source of text-based data crawled by machine learning Python programming language conducted from October 2018 until September 2019. The following data processing technique were consisted of three processes: data filtering, classification, and visualization of data collected through text mining. The results of this data processing were visualized via SNA (social network analysis), a technique for analyzing relationships between individuals through a collection of relationships or ties between nodes to establish the pattern of relationships between social actors ( Williams & Durrance, 2008). This study used SNA to visualize the network of conversations and hashtags produced by content creators (actors). These processes are part of a digital research method that can filter and verify digital data that are varied, dynamic, and interconnected ( Rogers, 2015).

Specifically, data processing in this research underwent data filtering, classification, and visualization to produce contextual digital data findings that can visualize findings objectively. The filtering process took place in two stages. In the first stage, filtering takes place by classifying conversations into irrelevant, unstructured, and structured conversations and eliminating irrelevant conversations. This process also collected the conversation metadata, such as date, time, account, and hashtags. The first filtering process was carried out on the Microsoft Excel application.

In the second stage, filtering was conducted by processing structured conversations. This process grouped conversations with similar meanings into categories of specific narrative types in separate Microsoft Excel documents. This produced a structured dataset of categorized conversations.

The following process is data classification, which was divided in two stages. In the first stage, similar conversations (also known as structured datasets) were grouped based on specific predetermined attributes, after which the categorization of conversations based on these attributes was grouped again based on the exact attributes. Furthermore, unstructured conversations underwent processing through machine-learning word clouds to produce word cloud visualizations. The resultant keywords were also verified to identify the meaning and context of the conversation, which are helpful in describing the research findings.

The final process was data visualization, which takes three forms: timeline visualization (chronology of conversations about the research topic), graphical visualization (distribution of hashtags), and SNA visualization (informal networks and hashtags that connect actors through SNA).

### Ethical approval

This research was reviewed for ethics clearance by the Universitas Pembangunan Nasional Veteran Jakarta’s internal research ethics commission and authorized by the Vice Chancellor for Academic Affairs at the University, numbered 3/UN61/PT.01.06/2022, dated April 1, 2021. The request for ethics clearance was made later after data collection with the research team’s extensive data method. Given that using data from personal accounts on social media, in this case, Twitter, also requires ethical conformity, we have proposed ethical clearance for this data requirement and received the approval on April 1, 2021.

## Setting the context: The emergence of the #ReformasiDikorupsi and #TolakOmnibusLaw movements

In 2019, the Indonesian Parliament ( *Dewan Perwakilan Rakyat,* DPR), on the recommendation of the executive, amended Law No. 19 of 2019 concerning the KPK Law (KPK Law) and discussed the Draft Criminal Code (RKUHP). Many elements in civil society view the revision of the KPK law and the discussion of the RKUHP as betraying the ideals of the 1998 Reformasi (Reformation) when the economic and political crisis finally brought mass protests and ousted Suharto, the president of 32 years. Many believe revising the KPK Law will weaken the Commission by reducing several of its powers to investigate corruption. At the same time, the RKUHP has the potential to limit the rights of citizens.

This decision was opposed by many elements of society, especially students. Tens of thousands of students in almost all cities flooded the streets in September 2019 with seven demands, including the cancellation of the revision of the KPK Law. They also urged the DPR to immediately pass the Bill on the Elimination of Sexual Violence and the Domestic Workers Protection Bill. Academics in the country and abroad also supported the student movement through an open letter stating their rejection of the revised KPK Law.

Following the escalation of protests in various cities, many experts suggested that the president immediately issue a Government Regulation Law Substitute as a solution. The pressing urgency was increasingly felt due to the wave of demonstrations, which were increasingly considered unstoppable. The action, which was triggered by public disappointment, immediately turned to anger after two students in Kendari, Southeast Sulawesi, La Randi, and Muhammad Yusuf Qardhawi, were killed by a police projectile during a demonstration ( Gabrilin, 2019). Thousands of others were arrested without a valid reason, and journalists were victimized by police brutality according to the recapitulation of the Indonesian Journal Alliance (AJI) recorded 14 cases of violence against journalists in various regions including Jakarta, Maksaaar, Palu and Jayapura ( Madrim, 2019). In this action, students had broad sympathy from various national and international groups, especially for the victims and in light of the severe repression of students. This sympathy resulted in support in the digital space, in this case, on social media. Public figures with large followings on social media, such as singer Ananda Badudu, also expressed sympathy and even invited the public to help and show solidarity. In his tweet, Badudu invited anyone who felt that the students’ demands were justified to help students in action in front of the DPR Building. Moreover, Badudu helped raise funds on the Kitabisa citizen funding platform to help with food and student logistics. Badudu was later arrested by the police as a result ( Persada, 2019).

In parallel, movement mobilization occurred both offline and online. When repression strengthens against the offline movement, the online movement in social media strengthens simultaneously. For example, the tweet from the Center for Anti-Corruption Studies Universitas Gadjah Mada @PUKAT_UGM, which became the top tweet #1, created a thread that briefly and quickly explained the problems in the Job Creation Bill, including the process, method of formation, and contents. The thread helps to understand the main problems of the Law in systematic and easy-to-read by common public.

Several students who took part in the action stated that their attitude was also extended to social media by using their accounts and delivering unique messages ( Adam, 2019). With public pressure on the government in response to various repressions over the student demonstrations, President Jokowi promised to delay revising the bills, particularly the amendments to the KPK Law. One year later, the government made a new policy that once again invited a strong reaction from the public, including university students. President Jokowi initiated an Omnibus Law compiling approximately 76 laws in 1,200 articles to avoid overlapping rules to make a less complex and more favorable environment for investors to enter Indonesia. However, drafting this law seemed hasty, without extensive consultation with the public. The president targeted the passage of this policy within 100 days. This raised public doubts, especially regarding the interests of several elite oligarchs in the current government, as stated by Wijayanto, the government’s motivation in passing the Job Creation Law quickly was because there are common interests, namely political economy among the oligarchic elite ( Dzulfaroh, 2020). The participation of young people, especially students, on social media in opposing the labor law is also widely reported through the mass media. As reported by Kompas, the biggest newspaper in Indonesia, the wave of rejection of the omnibus law has become a trending topic on Twitter. Their report is based on observations through social media analysts, and several hashtags dominate the conversations of netizens on Twitter, such as #TolakOmnibusLaw. While the conversation map on Twitter shows an exciting pattern, dominated by counter omnibus law clusters, even accounts from academics, BEM (Universitas Indonesia student union), NGOs, activists, and K-popers unite to support each other #MosiTidakPercaya and #DPRKhianatiRakyat. While the conversation map on Twitter shows an exciting pattern, dominated by counter omnibus law clusters, even accounts from academics, BEM, NGOs, activists, and K-popers unite to support each other ( Mulyana, 2022). CNN Indonesia also reported that there were many rejections of the omnibus law, such as “Failure of the Omnibus Law Echoing in Social Media, Support Action on the Road” or “The Beginning of the Omnibus Law Protest to Noise on Social Media” similar to the Kompas Media coverage. CNN Indonesia emphasized that the action of rejecting the omnibus law not only directly participated in the demonstration, but the demonstrators also echoed on Twitter social media by highlighting popular hashtags such as #GagalkanOmnibusLaw.

This bill was passed in a plenary meeting of the DPR on October 5, 2021. A day after the passage, simultaneous demonstrations involving tens of thousands of people were held in at least 18 provinces of Indonesia on October 6–8. The police again resorted to violence and repression, resulting in chaos and many victims, including high school and vocational high school students. Amnesty International Indonesia reported at least 51 videos showing 43 violent incidents in 15 provinces between October 6 and 10, 2021. In these videos, the police are seen brutally using blunt objects to disperse demonstrations, injuring at least 402 people and arresting 6,658 demonstrators ( Saputra, 2020).

In this protest, the participants of the demonstration were not predominantly students but included several others, including workers and activists. Labor was involved in large numbers, dominating the organizations in various regions of Indonesia. Students were less involved in numbers as extensively as the previous year Rahmah *et al.* (2020). This is also partly due to the COVID-19 pandemic that hit Indonesia in March 2020, imposing limitations on the recruitment and mobilization of protest movements. However, the pandemic has conversely afforded more excellent and broader online recruitment and mobilization opportunities. However, student organizations still voiced their support for the passage of the law through social media, such as the Student Executive Board of Universitas Indonesia (BEM UI). This organization has been opposed to ratification of the Job Creation Law from the beginning because considered to be able to harm the people. Not only through tweets but also, BEM UI said that the Job Creation Law since it was mentioned in the initial speech of the President of the Republic of Indonesia, reaped many problems, many rejections caused by product defects both ‘formally’ and ‘materially’.

## Discussion: Data analysis and findings

In this section, we will show the expansion of the digital movement on social media, which involves youth elements previously considered apolitical, such as K-Poppers. We collected data and information presenting the dominant narratives of the groups of young people involved in both movements. We divide the period into (1) the #ReformasiDikorupsi event, and (2) the #TolakOmnibusLaw event.

## The #ReformasiDikorupsi movement

In the #ReformasiDikorupsi movement, students started to build and mobilize support and participation through social media at least two weeks before any action took place. The hashtag #ReformasiDikorupsi first appeared on September 16 at 00:00:50 West Indonesia Time (WIB). Before that, the hashtag #RKUHPNgawur had already appeared on September 1 at 19:20:55 WIB.

Our study found the four most used hashtags: #ReformasiDikorupsi, or Corrupted Reform (18,460 lines); #TolakRKUHP, or Reject KUHP (53,922 lines); #RKUHPNgawur, or Inconsequent KUHP (1,099 lines); and #MahasiswaBergerak, or Students Move (53,922 lines). Hashtag #ReformasiDikorupsi shows that #Mahasiswa (college students) dominated the conversation. This shows that the most active or widely discussed segment of the youth groups involved in the movement were students.

**Figure 1.**
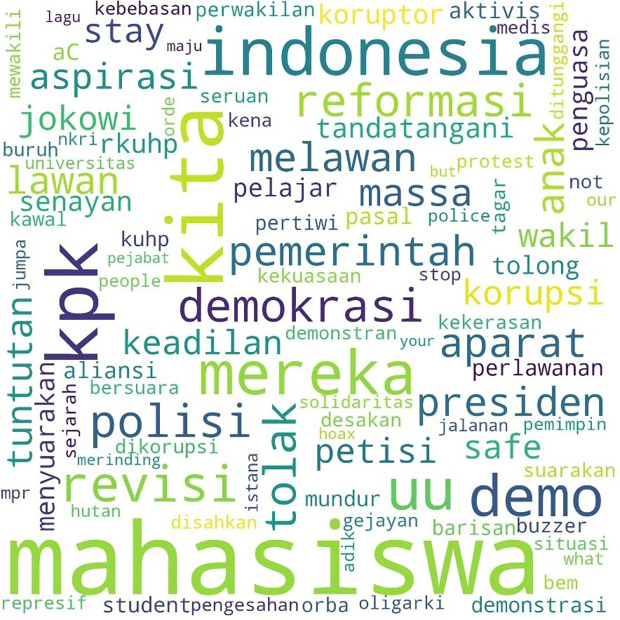
Word cloud for #ReformasiDikorupsi.

In Figure 1, #Students is the most talked-about term, showing students as the main subject in the movement. Student entities are described as #us, or us, and the targets of criticism as #them or them. Those who fall into the “our” category in this movement besides students are #pelajar or secondary students, #labor or labor, and #activists or activists. Several student entities were also discussed, including #BEM, the executive student organization in universities, and #gejayan, which refers to a student mobilization initiative in Yogyakarta. Meanwhile, tweets are addressed to parties such as #Jokowi, #President, #Government or government, #penguasa or rulers, #representasi or representatives, #polisi or police, and #buzzers, namely the influencers on social media who create narratives that defend government policies. The criticisms and concerns raised are the most common conversations, such as #KPK, Indonesia, #Democracy, #reject or reject, #revision or amendment, #UU or Law, and #korupsi or corruption.

**Figure 2.**
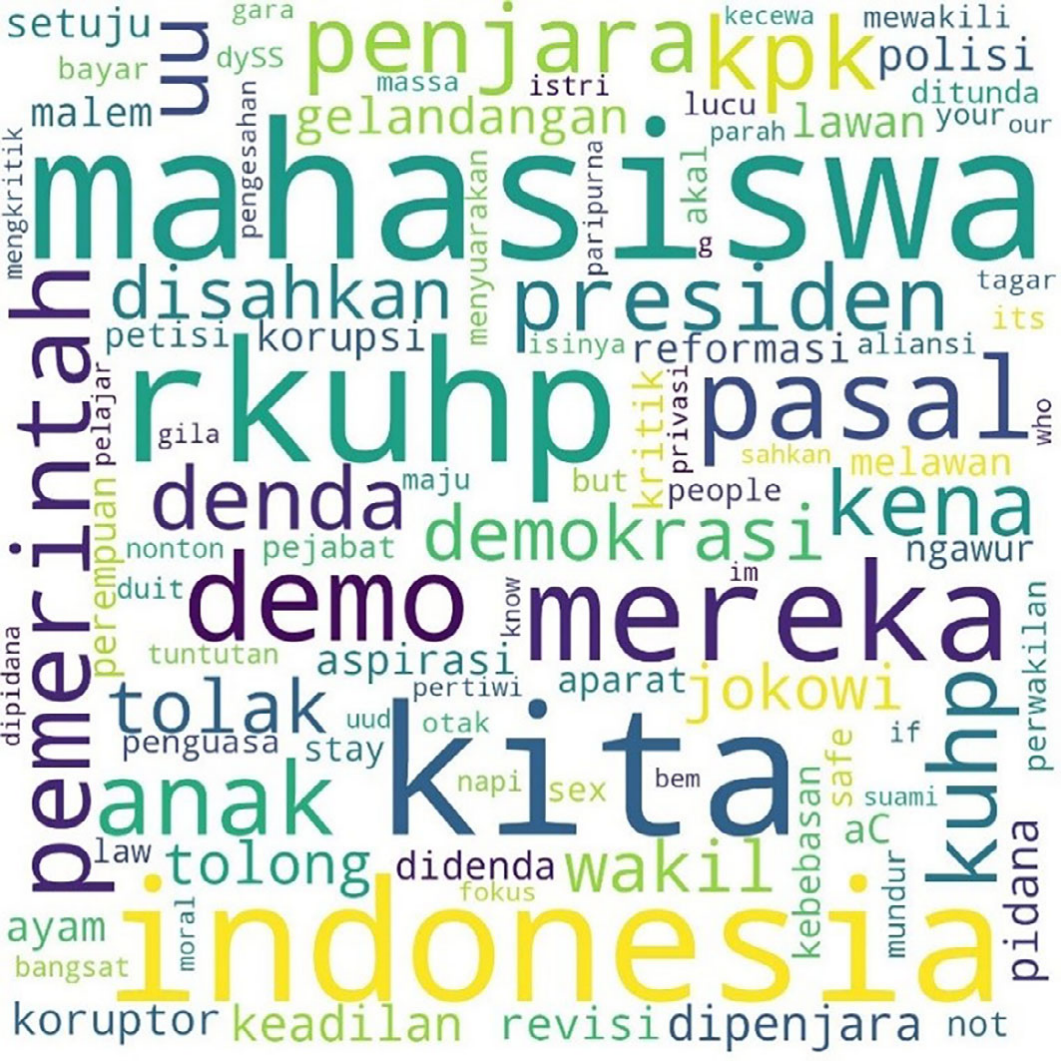
Word cloud for #TolakRKUHP.

Similarly, the pattern for the hashtag #TolakRKUHP also shows students as the most talked-about hashtag. #Indonesia and #RKUHP or the Criminal Law draft were also extensively discussed in the movement, and as the previous figure shows, the binary opposition between them and us also existed. Figure 2 shows the pattern.

The movement more specifically inspired the involvement of students with the hashtag #MahasiswaBergerak. The hashtag was first mentioned on September 6, 2019, at 03:49:07 WIB, and was involved in 53,922 lines of conversation. The picture below shows these conversations on Twitter.

**Figure 3.**
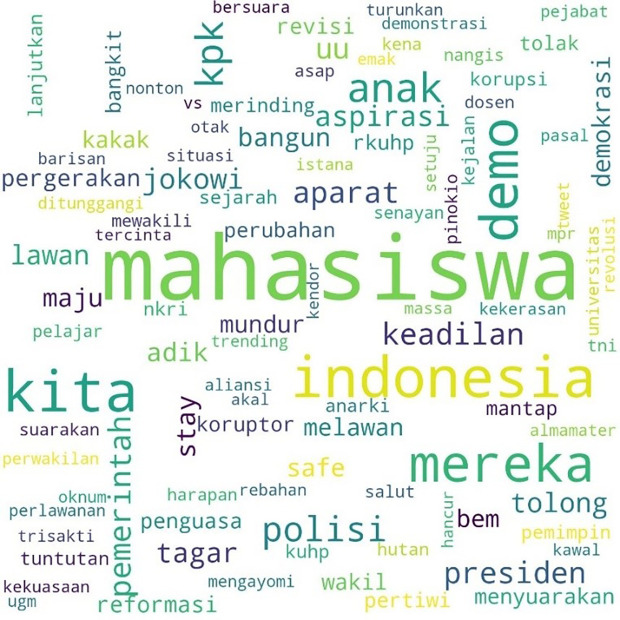
Word cloud for #MahasiswaBergerak.

In Figure 3, #student and #demo, an abbreviation for demonstration, were the two most common words. The rest, as in the two previous hashtags conversations, show the position of “us” and “them” in this widespread movement, and the form of criticism of the government and its apparatus.

In the conversations shown in the three figures, students were the predominant parties, and the conversations mainly concerned the revision of the Indonesian Criminal Law (KUHP), which held significant implications for the lives of young people, including threats to privacy, safety, and freedom of expression. The movement also echoed the protests for revision of the Anti-Corruption Law, and the readings find concern over this issue in the movement. What links these two concerns are the students’ assessment of a decline or setback in Indonesian reformation (reformasi) and democracy in general. The criticisms were addressed to the government, represented by #Jokowi as president and #DPR as parliament, and the state apparatus involved in legal reform such as #polisi or police.

In social media, parties who support this policy amendment also build a narrative. In our dataset, hashtags of those supporting the amendments to the KPK Law include #KPKcengeng or whining over the KPK, #belistepjokowi or trust Jokowi steps, and #revisiuukpkfornkri or revising KPK Law for the unitary state of Indonesia. The number of conversations using this hashtag is much smaller than those rejecting the law revision. The hashtag #kpkcengeng, for example, only appeared in 1,503 conversations. In comparison, #beliefstepjokowi appeared in 2,572 conversations, and #revisiuukpkfornkri appeared in 739 conversations, in strong contrast with the four hashtags against the amendments to the law described above. In addition, conversations supporting the revision of the law, most by buzzers’ accounts, also show trash hashtags or those that have no meaning other than to enliven the conversation, such as tribal hashtags like #sunda, #betawi, and #baduy, and hashtags whose relevance to this movement is not clear, such as #bakso and #biru.

## The #TolakOmnibusLaw movement

Our readings for the #TolakOmnibusLaw show some unique differences from the #ReformasiDikorupsi movement. The four most used hashtags in this movement are #tolakomnibuslaw (425,461 lines), #GagalkanOmnibusLaw (120,537 lines), #JegalOmnibusLaw (21,516 lines), and #JegalSampaiGagal (36,848 lines). In this movement, students did not dominate the conversation, as many more different groups of society were involved, including mostly labor groups.

**Figure 4.**
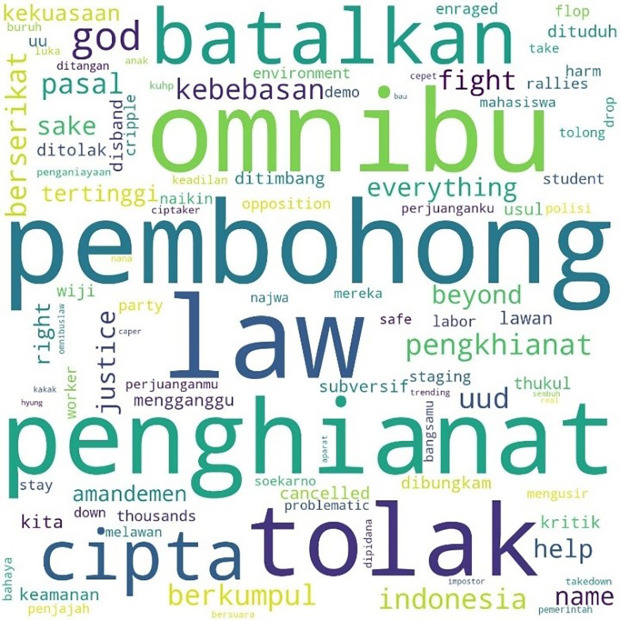
Word cloud for #TolakOmnibusLaw.

The most mentioned hashtag, #TolakOmnibusLaw, first appeared on October 6, 2020, at midnight. In Figure 4, we can see that the words #student and #mahasiswa were less widely used than words such as #pembohong (liar), #penghianat (traitor), or #Omnibus. This shows that the conversation was dominated by the substance of the criticism and did not focus on the actors as in #ReformasiDikorupsi. Apart from #Omnibus, #liar, and #traitor, criticism was expressed in a strong language such as #reject, #annul, #freedom, #fight, #right, and #justice.

**Figure 5.**
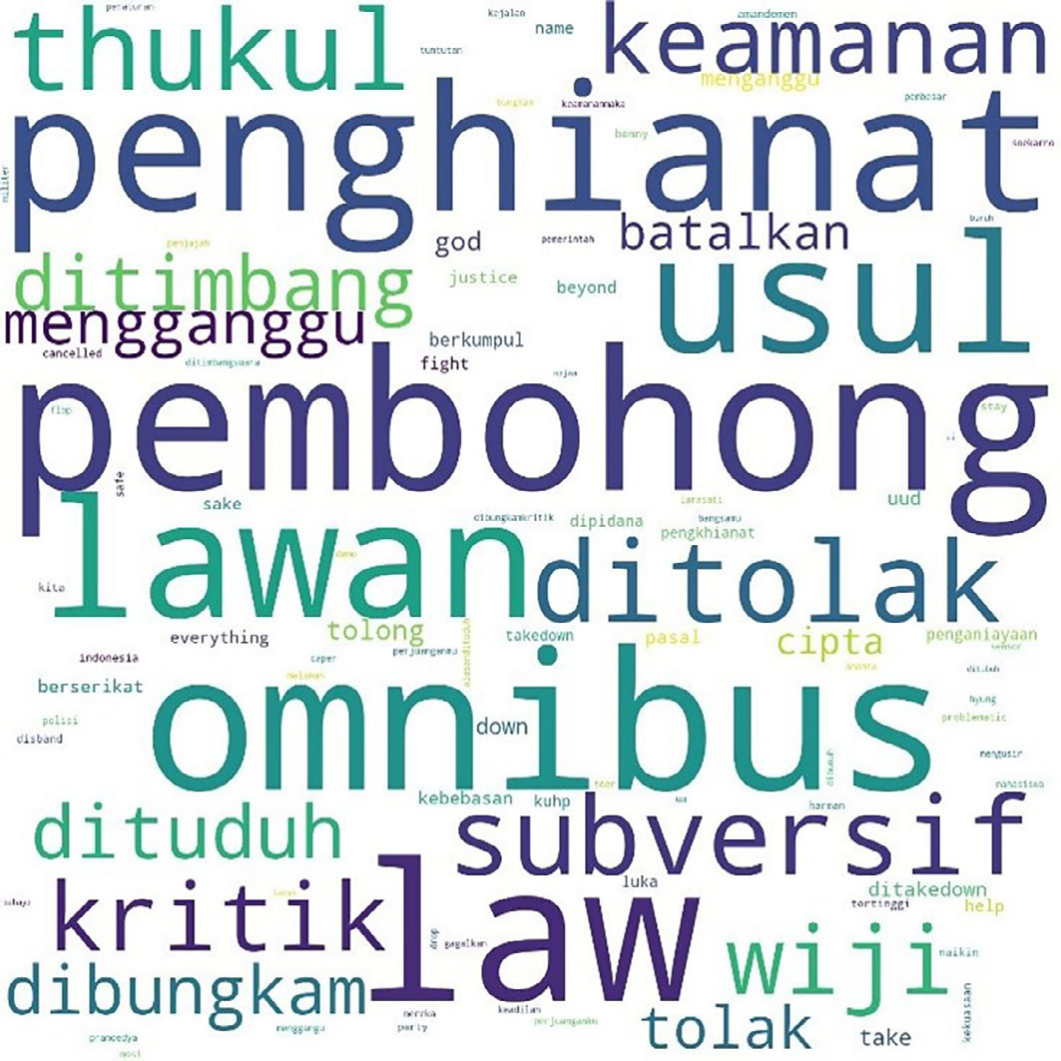
Word cloud for #GagalkanOmnibusLaw.

The readings for #GagalkanOmnibusLaw involved 120,537 lines of conversation as shown in Figure 5, initially mentioned on October 6, 2020, at 06:59:59. Similar to #tolakomnibuslaw, the lines were dominated by words such as “liar,” “traitor,” and “omnibus.” Other words included #keamanan or “security,” #usul or “initiative,” #subversif or “subversion,” #dituduh or “accused,” #dibungkam or “silenced,” #ditimbang or “considered,” #mengganggu or “disturb,” and #batalkan or “cancel.”

**Figure 6.**
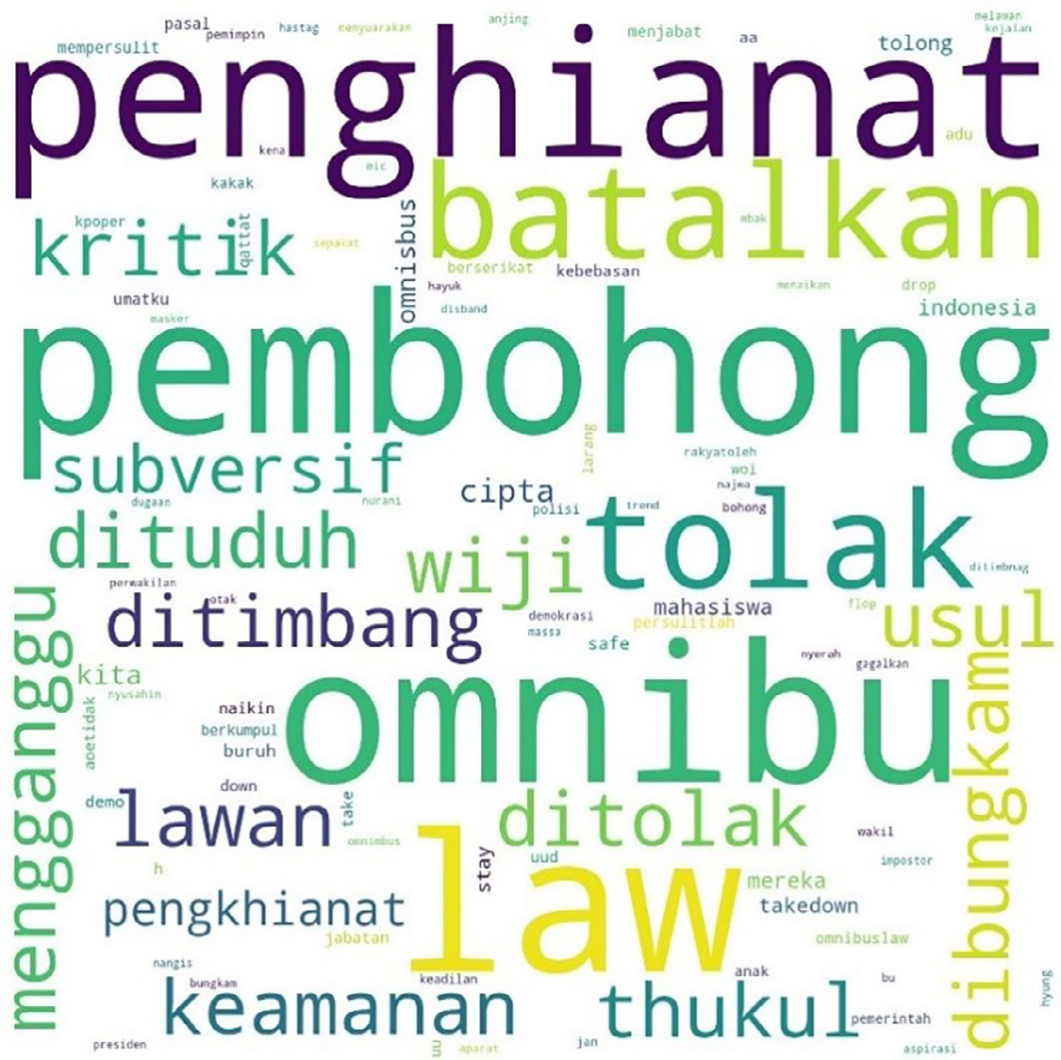
Word cloud for #jegalomnibuslaw.

We found a similar pattern for #jegalomnibuslaw, also first appearing on October 6, 2020, at 21:59:57, as shown in Figure 6.

**Figure 7.**
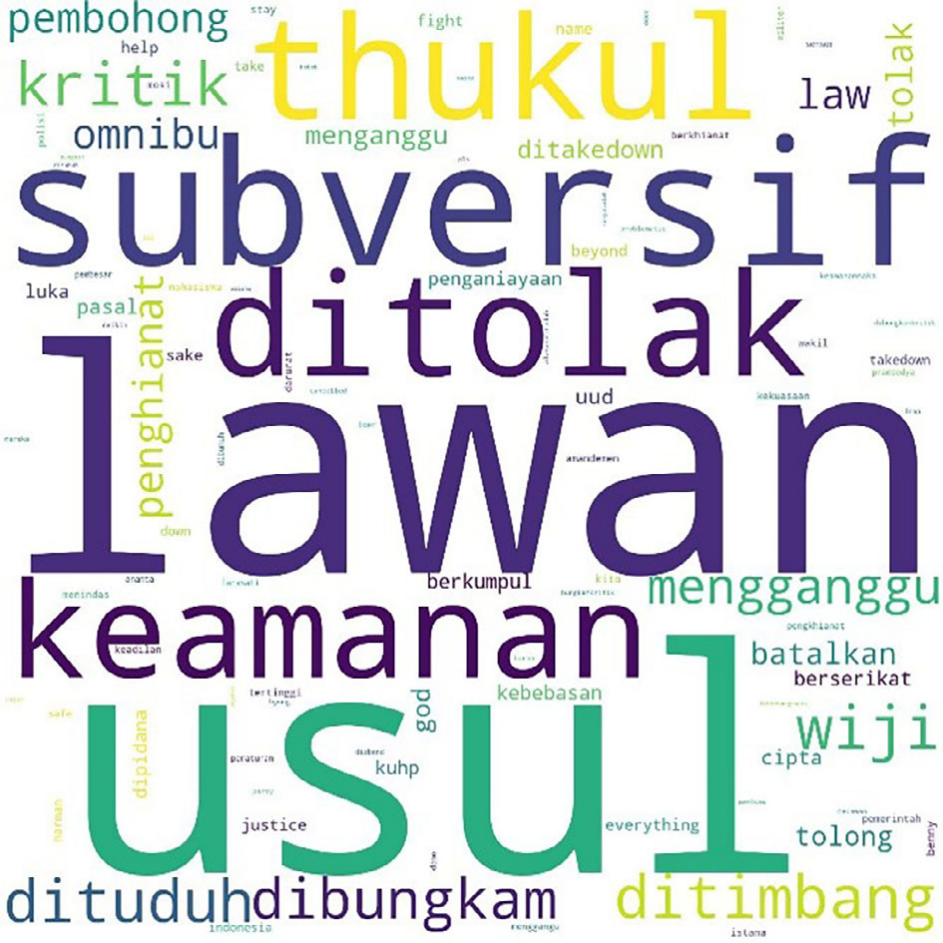
Word cloud for #jegalsampaigagal.

Two days after the initial mention of #jegalomnibuslaw, another hashtag, #jegalsampaigagal was first mentioned at 00:00:16. In Figure 7, we see that conversations with this hashtag were dominated by the word “fight” (lawan), a word often quoted from a poem by Widji Thukul, a pro-democracy activist who disappeared in 1997 for his “subversive” and critical poems. The words Widji, Wiji, Thukul, and #lawan were used repeatedly and dominated conversations during the Tolak Omnibus Law movement.

**Figure 8.**
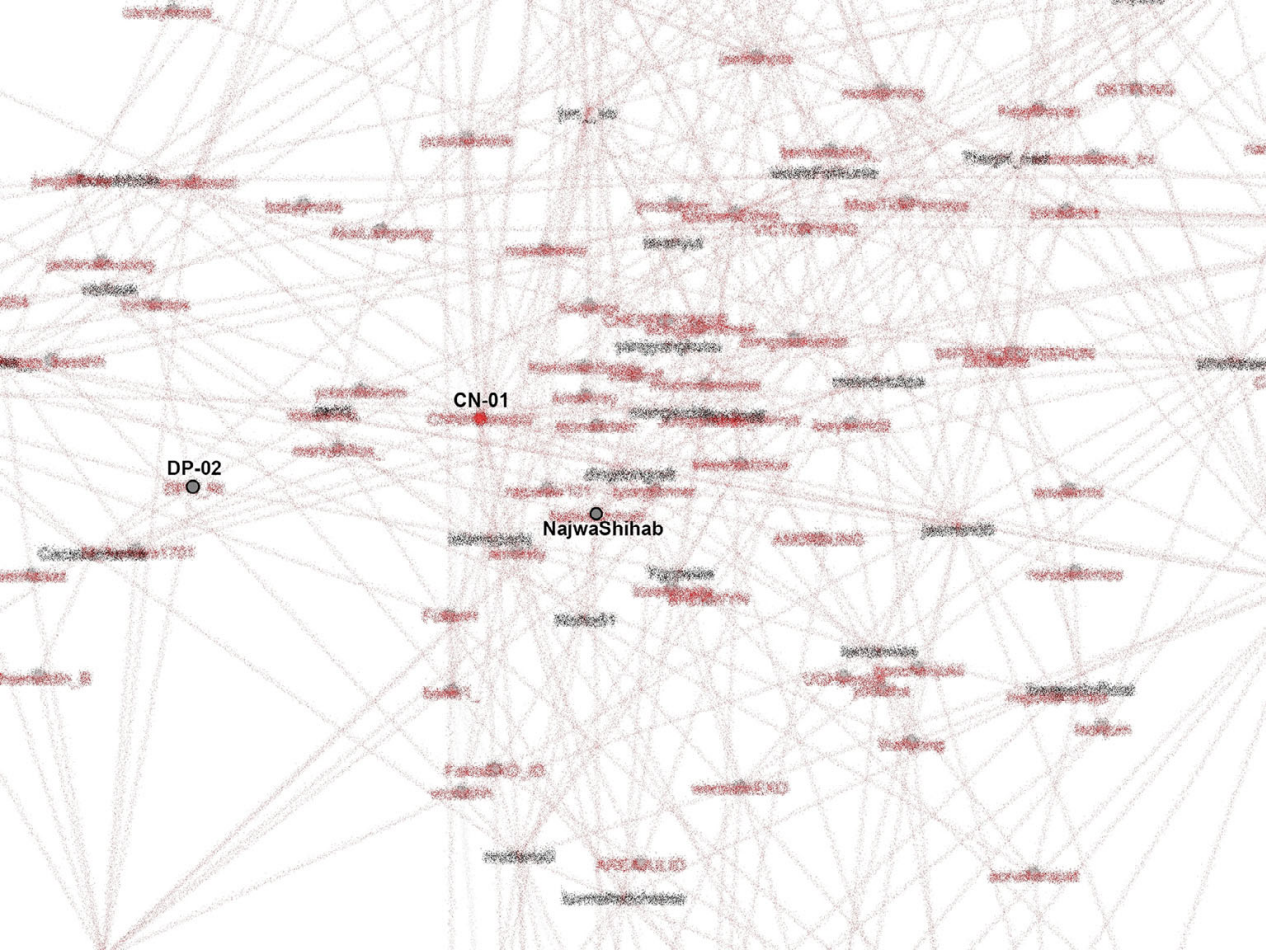
SNA of accounts in #TolakOmnibusLaw movement (anonymized). CN-01: @CNNIndonesia, DP-02: @DPR_RI.

The active roles of K-Poppers in the movement indicated youth involvement. K-Poppers are a community of young people. Generally, Generation Z means people who were born between 1997 and the 2000s and are devoted fans of popular Korean products, including movies and songs. The top hashtags first appear on accounts representing K-Poppers that not only use the names of Korean artists or popular Korean words but whose stories and main pages generally relate to K-Pop. K-Poppers’ intense involvement in this movement ( Mulyana, 2022) can be observed from the SNA shown in Figure 8.

K-Poppers, academic accounts, BEM, NGOs, and activists presented the main opposition to the Omnibus Law. These accounts united and supported each other to form a large cluster ( Fahmi, 2020), showing the significant role of K-Poppers as a solid and influential group in the #TolakOmnibusLaw movement on Twitter. Drone Emprit’s research identified an extraordinary increase in conversations on Twitter after the Omnibus Law was passed ( Fahmi, 2020). K-Poppers’ presence can be seen in the number of profile photos in the conversation about the Omnibus Law ( Fahmi, 2020).

Our dataset includes many accounts with K-Pop terms and names of artists. The most retweeted can be seen in Figure 8. We identify K-Popper accounts using Korean terms or elements of idol and group names in the Twitter account name, as well as profile photos and tweets discussing various K-Pop matters.

**Figure 9.**
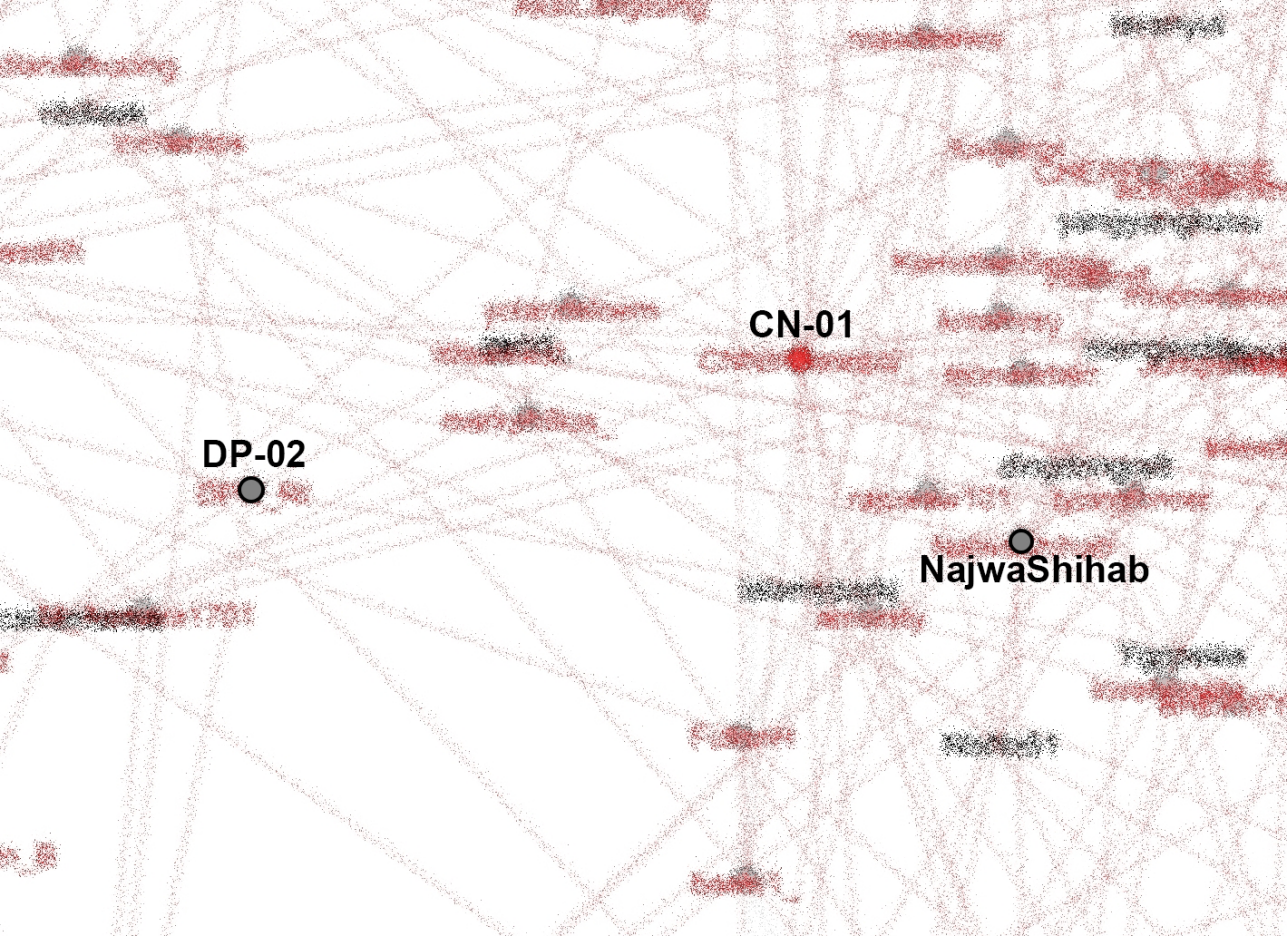
The center of the SNA in #TolakOmnibusLaw movement (anonymized). CN-01: @CNNIndonesia, DP-02: @DPR_RI.

From our SNA mappings, we identified many K-Poppers accounts that were involved in the #TolakOmnibusLaw movement. If we look more specifically at the SNA in the middle position, which occupies the most central position in the movement, we obtain Figure 9.

**Figure 10.**
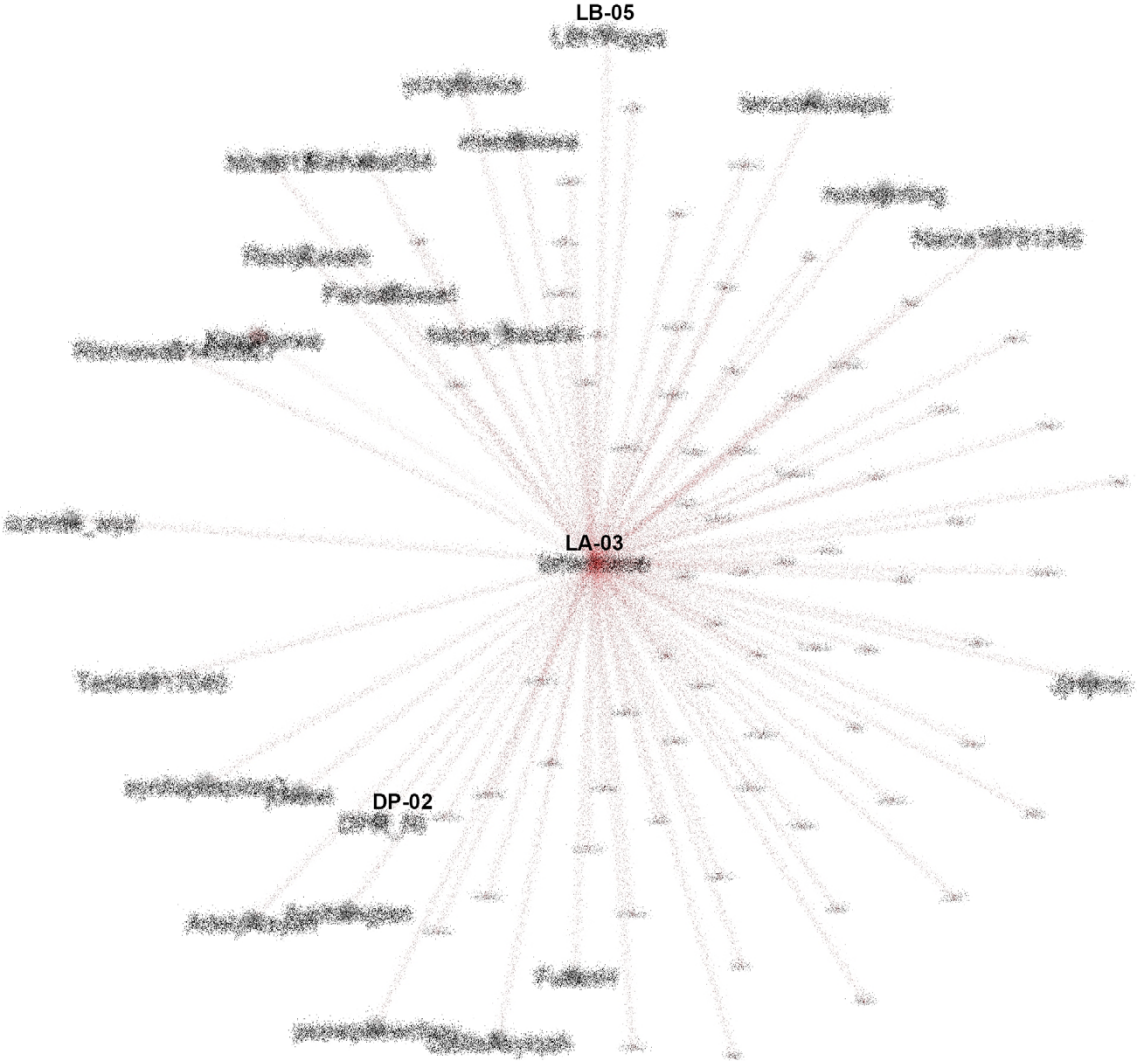
SNA of the most referenced account during #TolakOmnibusLaw (anonymized). LB-05: @LBHYogya, DP-02: @DPR_RI.

To see how important the role of K-Poppers is in the movement, we looked at the most referenced account (LA-03), and saw that for #TolakOmnibusLaw, this account was followed and retweeted by various other accounts, including the Indonesian Parliament (@dpr_ri) and public figures such as Najwa Shihab (@najwa_shihab), a famous senior journalist and TV host, NGOs such as LBH Yogyakarta (@LBHYogya), and mainstream media like CNN ( Figure 10).

**Figure 11.**
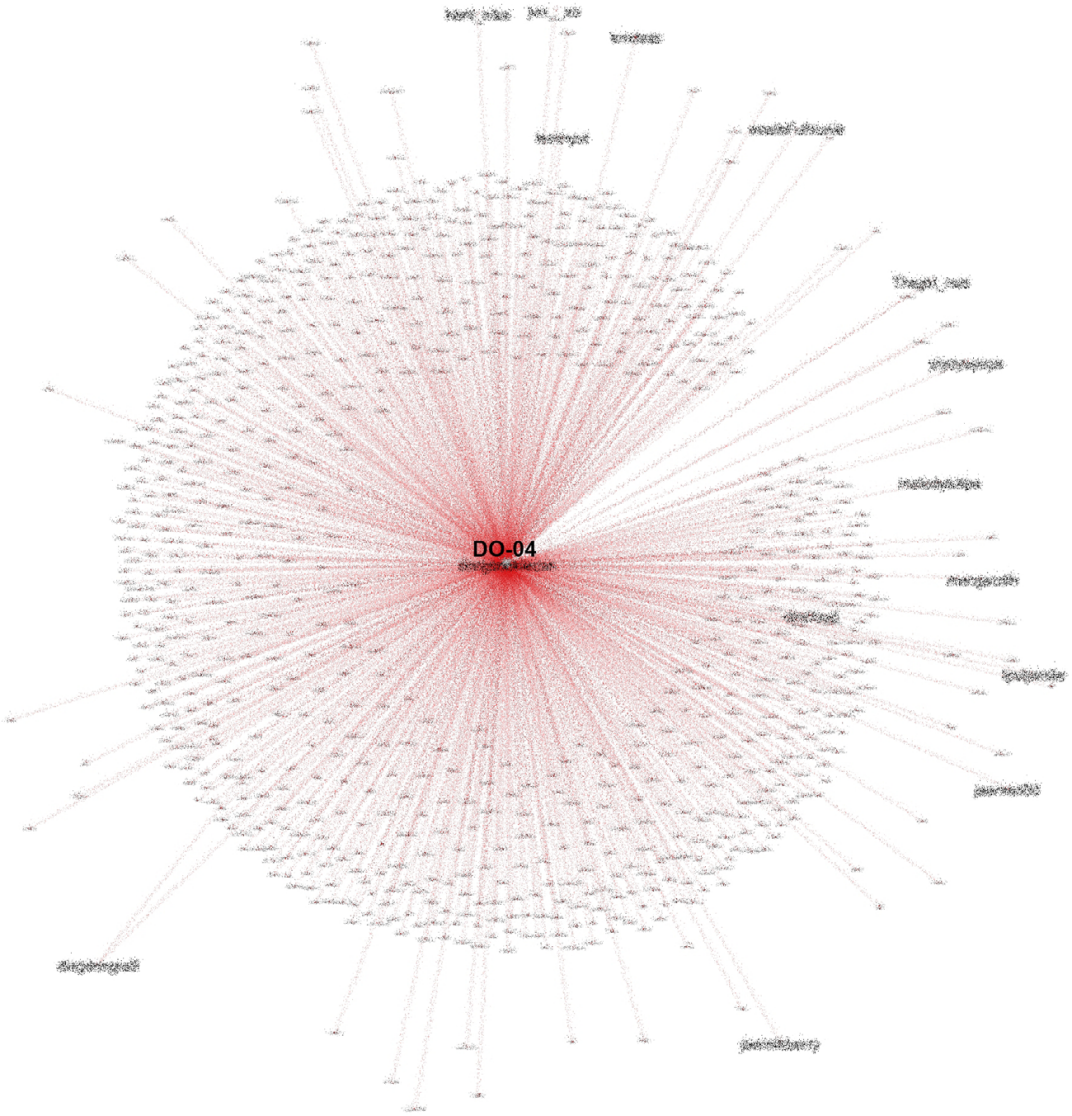
SNA of another heavily referenced account during #TolakOmnibusLaw (anonymized).

Another account (DO-04) that is also heavily referenced in conversations on #TolakOmnibusLaw also had many followers and was often retweeted, especially by fellow K-Popper accounts, as shown in Figure 11.

From the information above, we see the vital role of young people in these two movements which present criticism towards the state policies. This study aims to provide a snapshot and explanation of the political participation of young people in digital activism, both online and offline, criticizing government policies. Furthermore, we observe different patterns for the two movements. In #ReformasiDikorupsi, students dominate the protests. The repression and violence of the security forces ultimately led to digital activism that focused on topics related to students.

Conversely, in the #TolakOmnibusLaw protests, students, along with other elements of society, especially workers, took to the streets and protested state policies that were not transparent and participatory. Digital activism started to grow when offline action started ( Rahmah *et al.*, 2020). This was also conditioned by the COVID-19 pandemic, causing students to participate in smaller numbers than the previous year. Although young K-Poppers were involved in #ReformasiDikorupsi, their role in the #RejectOmnibusLaw movement was more active and significant from the beginning.

The extensive data analysis method we chose in this study has several weaknesses. First, quantitatively using big data, we can only analyze patterns and interpret them to get answers to research questions. However, we needed the opportunity to investigate in depth and verify what motivates Twitter users to engage in digital activism and their experiences in that activism. The second, which is also very principal, is the limitation in verifying the owners of these accounts. In this case, we focus on the criteria of young people who are, among other things, limited by age, but this is precisely what needs to be verified from the hundreds of thousands of accounts included in our analysis. However, two of the students on our team involved in this study conducted their studies with a specific focus on investigating specific accounts. The first research focuses on the role of k-poppers in the #TolakOmnibusLaw movement, and the second is the role of pro-democracy activists in mobilizing the #TolakOmnibusLaw movement.

## Conclusion

Throughout this article, we provided an overview and understanding of the phenomenon of youth involvement in two critical movements in Indonesia since the Reformation began in 1998, namely the 2019 #ReformasiDikorupsi and the 2020 #TolakOmnibusLaw. Specifically, we focused our investigation on youth engagement in digital-based social movements on Twitter. Using data mining techniques, we confirmed that youth groups, especially students and millennial K-Poppers, actively participated in the movement and mobilized it by generating hashtags and expanding conversations on Twitter. The emergence of a digital society allows for mutual connection and expansion of the venue for freedom of expression as well as a form of active participation in political developments at the state level. Thus, this group of youth provided a valuable contribution to the development of democracy in Indonesia. Although the activism of young people on social media is massive, this movement is inseparable from the large-scale demonstrations that took place offline in many large cities in Indonesia. In this case, the two movements, online and offline, influenced each other. The online medium constituted a recruitment space for building, framing, and mobilizing the offline movement. As Cammaerts (2015) highlights, activism in social media can involve three activities: mobilization and recruitment, channeling communication, and direct action opposing the government’s policies. Mobilization and recruitment took place before and during the offline movement. One example is #gejayanmemanggil, or gejayan calling, referring to Gejayan, a meeting point in Yogyakarta where students gather and consolidate offline. As alternative communication channels, social media facilitate activists’ self-mediation by distributing goals and framing movements more efficiently and enable activists to transmit text and visual discourse, as well as afford opportunities for communities and subordinate groups to build alternative collective identities. Direct actions were communicated effectively in social media, helping construct the binary opposition of “Us” (#Kita) and “Them” (#Mereka).

Conversely, the repression of demonstrators, especially university students and secondary school students, strengthened the movement with broader forms of solidarity and mobilization. As in Hong Kong ( Wong, 2021), young people’s reliance on social media to receive and share news and information on public affairs created an echo chamber effect that significantly divided social media-engaged youth from the rest of the population.

The massive size of this movement, online and offline, of course, cannot be separated from the context of its emergence. In this case, illiberal democratic practices, such as non-transparency and exclusivity in policy making by certain elites, policy inconsistencies, or responses to criticism with violence and repression, are alarms for young people to express their concerns and voice their aspirations. Diaz, a student who joined in an offline demonstration and later went viral in social media for a tweet on cosmetic brands suitable for a demonstration, stated in a media interview: “Yesterday’s demo proved many things, but what should be highlighted the most is that our situation means that it is severe. That is why students protested in large numbers” ( Adam, 2019). This statement shows the concern of young people for the socio-political conditions in Indonesia, and their participation in voicing these concerns is a separate part of the meaning of their political identity.

## Data Availability

The underlying data to this research cannot be shared due to the ethical and copyright restrictions surrounding social media data. The Methods section also contains detailed information to allow replication of the study. Any queries about the methodology should be directed to the corresponding author.
