# Supplementary material for: Youth political participation and digital movement in Indonesia: the case of #ReformasiDikorupsi and #TolakOmnibusLaw
Source: F1000Res. 2024 Apr 25;12:543. Originally published 2023 May 24. [Version 3] doi: 10.12688/f1000research.122669.3 (PMC11109537; doi:10.12688/f1000research.122669.3)
Supplement: Peer review history

# Reviewer report: Reviewer response for version 3

**DOI:** 10.5256/f1000research.165370.r269504

**Published:** 2024-05-09

**Authors:** Oluyinka Osunkunle

**Affiliations:** 1 University of Fort Hare, Alice, Eastern Cape, South Africa

The authors have attended to the comments given to improve their discussions and ensure a better quality of the paper.

 There is now clarity under Literature Review with key issues discussed well-presented unlike before when they were mixed up. So this section is better now

 Also, there is now clarity and well demarcated discussion on Study Findings 

 Overall, the Paper sounds better in terms of presentation, organisation and clarity.

Is the work clearly and accurately presented and does it cite the current literature?

Yes

If applicable, is the statistical analysis and its interpretation appropriate?

Yes

Are all the source data underlying the results available to ensure full reproducibility?

Yes

Is the study design appropriate and is the work technically sound?

Yes

Are the conclusions drawn adequately supported by the results?

Yes

Are sufficient details of methods and analysis provided to allow replication by others?

Yes

Reviewer Expertise:

Political Communication, Media and Society, new/social media efficacies and Stakeholder Relations

I confirm that I have read this submission and believe that I have an appropriate level of expertise to confirm that it is of an acceptable scientific standard.

# Reviewer report: Reviewer response for version 3

**DOI:** 10.5256/f1000research.165370.r269503

**Published:** 2024-05-08

**Authors:** M. Zaenul Muttaqin

**Affiliations:** 1 Department of Public Administration, Cenderawasih University, Jayapura, Papua, Indonesia; 2 Doctoral Student of Public Administration, Universitas Negeri Makassar, Makassar, South Sulawesi, Indonesia

I have read the authors' revisions and for this reason I recommend this article to be read widely, especially by scholars with an interest in contemporary political communication research.

 Best regards

Is the work clearly and accurately presented and does it cite the current literature?

No

If applicable, is the statistical analysis and its interpretation appropriate?

I cannot comment. A qualified statistician is required.

Are all the source data underlying the results available to ensure full reproducibility?

Yes

Is the study design appropriate and is the work technically sound?

Partly

Are the conclusions drawn adequately supported by the results?

No

Are sufficient details of methods and analysis provided to allow replication by others?

Partly

Reviewer Expertise:

Policy Communication

I confirm that I have read this submission and believe that I have an appropriate level of expertise to confirm that it is of an acceptable scientific standard.

# Reviewer report: Reviewer response for version 2

**DOI:** 10.5256/f1000research.163213.r264563

**Published:** 2024-04-18

**Authors:** M. Zaenul Muttaqin

**Affiliations:** 1 Department of Public Administration, Cenderawasih University, Jayapura, Papua, Indonesia; 2 Doctoral Student of Public Administration, Universitas Negeri Makassar, Makassar, South Sulawesi, Indonesia

Thank you to the authors for working hard to complete all previous recommendations. I approve of this manuscript with its touch on explicit contributions to the field of knowledge, especially political science.

Is the work clearly and accurately presented and does it cite the current literature?

No

If applicable, is the statistical analysis and its interpretation appropriate?

I cannot comment. A qualified statistician is required.

Are all the source data underlying the results available to ensure full reproducibility?

Yes

Is the study design appropriate and is the work technically sound?

Partly

Are the conclusions drawn adequately supported by the results?

No

Are sufficient details of methods and analysis provided to allow replication by others?

Partly

Reviewer Expertise:

Policy Communication

I confirm that I have read this submission and believe that I have an appropriate level of expertise to confirm that it is of an acceptable scientific standard, however I have significant reservations, as outlined above.

# Response

**Published:** 2024-04-21

**Authors:** Sri Lestari Wahyuningroem

**Affiliations:** Universitas Pembangunan Nasional Veteran Jakarta, Indonesia

Thank you for the feedback. We have added two sentences on the contribution of this research to political science, as suggested by the Reviewer. The addition is on the last paragraph of the Introduction section.

# Reviewer report: Reviewer response for version 1

**DOI:** 10.5256/f1000research.134690.r191178

**Published:** 2023-08-17

**Authors:** Oluyinka Osunkunle

**Affiliations:** 1 University of Fort Hare, Alice, Eastern Cape, South Africa

**Contribution of Article to Knowledge**

 The paper no doubt contributes to knowledge in the field and especially looking at youth political participation in digital-based social movements.

**Introduction**

 The author/s gave a very good background to the study by looking at Internet and social media and how they have influenced the participation of youths inter alia student movements in digital democracy in Indonesia. The article also made reference to certain protests against corruption, and changes in democratic patterns by citing protests such as the #ReformasiDikorupsi movement in 2019 and #TolakOmnibusLaw movement in 2020. #ReformasiDikorupsi and also #TolakOmnibusLaw, which was a 2020 movement of students, trade unions, and civil society groups.

 This paper is unique and should contribute to knowledge in the sense that it focuses on the relationship between youth political participation, digital media, and the development of youth political identity while also analyzing young people’s experiences of participation in Indonesia, especially during the mass protests of #ReformasiDikorupsi in 2019 and #TolakOmnibusLaw in 2020.

 Also noteworthy is the fact that the paper highlights the role of young citizens not only as participants, but also core as actors who mobilized these protest movements to become so large-scale. Interestingly, the study also made reference to other youth political participations around the world as the Indonesia cases were discussed.

**Literature Review**

 The author/s have reviewed current/recent literature, which is a page and a half, which in my view, I deemed to be too little unless if the Journal allows it. Adding a paragraph of discussion on another key issue would do the paper a lot of good. Also, the author/s only had a single long discussion under Literature Review without any heading or subheading to show or demarcate the issues or literature being reviewed. Separating discussions under suitable heading/subheading helps to ensure clarity and better understanding to guide the readers. The author/s should revise and ensure this to improve the quality of the paper.

**Method**

 The study design is good as the paper/study has made use of suitable research method for data collection and analysis with a mixed-method approach employed to collect and analyze case study data by integrating complementary quantitative and qualitative approaches. The data considered or analysed in this study were digital conversations produced and mobilized by netizens on Twitter within the conversation period from October 2018, when the #ReformasiDikorupsi started, until the end of November 2019, after the #TolakOmnibusLaw movement ended. This research quantitatively used text mining, machine learning word clouds, and social network analysis (SNA) with specific computational configurations to produce quantified text data.

**Data Analysis/Findings/Discussion**

 I see that data was analysed but for the sake of the readers and to ensure clarity in this paper, the author/s need to clearly show us where data analysis, findings and discussions are in this paper. After a good discussion on ‘Methods’, the author/s went on to discuss ‘Ethical Approval’ and after this, there is a discussion titled ‘The emergence of the #ReformasiDikorupsi and #TolakOmnibusLaw movements’. The question here is, could this the beginning of the section on Data Analysis or Findings? Or is it focused on reviewing literature or just discussing about the movements under study?

 It therefore becomes very important for the author/s to properly demarcate the writings to ensure clarity. If the discussion after ‘Ethical Approval’ is Data Analysis or Discussion of Findings etc, then they should put a suitable heading/subheading there to show this to ensure clarity and also help the readers to have a good understanding of key issues discussed.

**Style and Clarity**

 There are no grammatical or spelling glitches and the author’s writing style is clear to some extent.

**Conclusion**

 The author/s have come up with a very good conclusion in relation to the study.

**Recommendation**

 The paper is well written and very relevant to contemporary society considering the several global cases of youth political participation in digital-based social movements. Documenting the use of the Internet and social media and how they have influenced the participation of youths inter alia student movements in digital democracy in Indonesia is noteworthy. There is evidence of good data analysis and conclusion too. In light of this, I recommend that the paper be accepted with minor correction – the author/s need to clearly demarcate / identify the Data Analysis section. They need to read through the paper again to ensure it is clean and well edited and that there is a good flow across the sections and headings therein.

Is the work clearly and accurately presented and does it cite the current literature?

Yes

If applicable, is the statistical analysis and its interpretation appropriate?

Yes

Are all the source data underlying the results available to ensure full reproducibility?

Yes

Is the study design appropriate and is the work technically sound?

Yes

Are the conclusions drawn adequately supported by the results?

Yes

Are sufficient details of methods and analysis provided to allow replication by others?

Yes

Reviewer Expertise:

Political Communication, Media and Society, new/social media efficacies and Stakeholder Relations.

I confirm that I have read this submission and believe that I have an appropriate level of expertise to confirm that it is of an acceptable scientific standard, however I have significant reservations, as outlined above.

# Response

**Published:** 2024-02-28

**Authors:** Sri Lestari Wahyuningroem

**Affiliations:** Universitas Pembangunan Nasional Veteran Jakarta, Indonesia

The focus was adjusted as suggested by the Reviewer. 

 The literature review has been shortened as suggested by the Reviewer. 

 We made clear the discussion into context and data analysis and findings sections. This is to make clear of the structure and focus as commented by the Reviewer. 

# Response

**Published:** 2024-02-28

**Authors:** Sri Lestari Wahyuningroem

**Affiliations:** Universitas Pembangunan Nasional Veteran Jakarta, Indonesia

Thank you for the comments and feedbacks from the Reviewer.

# Reviewer report: Reviewer response for version 1

**DOI:** 10.5256/f1000research.134690.r178191

**Published:** 2023-06-15

**Authors:** M. Zaenul Muttaqin

**Affiliations:** 1 Department of Public Administration, Cenderawasih University, Jayapura, Papua, Indonesia; 2 Doctoral Student of Public Administration, Universitas Negeri Makassar, Makassar, South Sulawesi, Indonesia

This paper raises a comprehensive theme, paying attention to political participation and youth digital movements in Indonesia. Overall, this study uses the right method to answer what is the research problem formulation. It's just that there is no clear focus. In every part of the writing, apart from lack of consistency.

**Abstract**

 The younger generation has always been at the forefront and made a significant contribution to social movements that are critical of state policies. The purpose of this research is to explore the relationship between youth political participation, digital media, and the development of youth political identity. The political identity of young people needs to be emphasized in the concluding part of this section.

**Introduction**

 There is no explanation regarding the platform that is the focus of research in the background section. The author can ensure the focus of his explanation, is it the role of the younger generation or the role of social media? In addition, this study describes too many problem formulations, so the authors lose focus. For that the author must explore a topic that is urgent.

 Answering this, will provide an explanation regarding the unclear structure and contribution of this research in a consistent and systematic manner.

**Literary Review**

 In this section, paragraph four, the statement that online activism has no substitute for offline activism needs to be clarified.

**Methods**

 The keywords used need to be explained by the author, so as not to confuse the withdrawal of the keywords used in this reader's research.

 In this section sufficient detail of the methods and analysis is provided to allow replication by others as it combines both quantitative and qualitative research for a comprehensive problem

**Results**

 The author needs to add a discussion section. Because the results of this study look partial without adequate focus

**Conclusion**

 In this section, the authors need to align with the objectives and results of this study

Is the work clearly and accurately presented and does it cite the current literature?

No

If applicable, is the statistical analysis and its interpretation appropriate?

I cannot comment. A qualified statistician is required.

Are all the source data underlying the results available to ensure full reproducibility?

Yes

Is the study design appropriate and is the work technically sound?

Partly

Are the conclusions drawn adequately supported by the results?

No

Are sufficient details of methods and analysis provided to allow replication by others?

Partly

Reviewer Expertise:

Policy Communication

I confirm that I have read this submission and believe that I have an appropriate level of expertise to confirm that it is of an acceptable scientific standard, however I have significant reservations, as outlined above.

# Response

**Published:** 2024-02-28

**Authors:** Sri Lestari Wahyuningroem

**Affiliations:** Universitas Pembangunan Nasional Veteran Jakarta, Indonesia

1. We’ve revised the introduction, main analysis and conclusion, to make it clear that the focus of this article is to look on the roles of young people in social movement through digital platform, and to discuss their engagement and mobilization that contribute significantly in the larger movement.

 We’ve revised the introduction, main analysis and conclusion, to make it clear that the focus of this article is to look on the roles of young people in social movement through digital platform, and to discuss their engagement and mobilization that contribute significantly in the larger movement.

 2. We've revised the Abstract as suggested. 

 3. We've revised the Introduction to be more focus as suggested by the Reviewer. 

 4. We've made the clarification as suggested. 

 5. Discussion was added to include the focus if the paper. 

 6. We've revised to align with objectives of the study.

# Response

**Published:** 2024-02-28

**Authors:** Sri Lestari Wahyuningroem

**Affiliations:** Universitas Pembangunan Nasional Veteran Jakarta, Indonesia

Thank you for comments and feedbacks from the Reviewer.
